# Parental Involvement in the Preoperative Surgical Safety Checklist Is Welcomed by Both Parents and Staff

**DOI:** 10.1155/2014/791490

**Published:** 2014-04-13

**Authors:** Martin T. Corbally, Eamon Tierney

**Affiliations:** ^1^Our Lady's Hospital for Sick Children, Crumlin, Dublin 12, Ireland; ^2^Paediatric Surgery, Royal College of Surgeons, Dublin 2, Ireland; ^3^King Hamad University Hospital, Busaiteen, P.O. Box 24343, Bahrain; ^4^Anaesthesia and Intensive Care, RCSI Medical University of Bahrain, Al Sayh, Bahrain

## Abstract

We involved the parents of paediatric patients in the first part of the three-stage WHO Surgical Safety Checklist (SSC) process. Forty-two parents took part in the study. They came to the theatre suite with their child and into the induction room. Immediately before induction of anaesthesia they were present at, and took part in, the first stage of the three-stage SSC process, confirming with staff the identity of their child, the procedure to be performed, the operating site, and the consent being adequately obtained and recorded. We asked parents and theatre staff later whether they thought that parental involvement in the SSC was beneficial to patient safety. Both parents and staff welcomed parental involvement in the WHO Surgical Safety Checklist and felt that it improved patient safety.

## 1. Introduction

The past thirty years have witnessed major technological advances in medicine and surgery that have in turn generated an expectation that all aspects of care should be delivered faultlessly and without negative consequence to the patient.

However the latent marriage of human error and systems failure continues to contribute to shortfalls in care delivery and this is especially evident in the continuing risks involved in anaesthesic and surgical practice. These risks are not insignificant and are reported to be as high as 22% in all surgical procedures, with an overall mortality approaching 1% [1–3].

Arising from this reported negative outcome, the World Health Organisation (WHO) introduced the Surgical Safety Checklist in 2008 to reduce the risk of adverse events during surgery. The checklist is in three parts consisting of

- an initial check (time in) by the anaesthetist and his assistant;
- a “time out” checklist before incision by the surgeon anaesthetist and nurse;
- a final check by the surgeon, anaesthetist, and nurse before the patient leaves theatre.

These checks are designed to ensure the following.

- The patient is the correct patient.
- The planned surgery is the correct surgery for the patient.
- The surgical site is marked on the correct side.
- All equipment, both anaesthetic and surgical, is available.
- The anaesthetist is prepared for any patient allergy, airway problem, and blood loss.
- Prophylactic antibiotics are given if considered appropriate.
- The surgeon has essential imaging displayed, performs the correct operation, and anticipates duration of surgery and blood loss.
- All specimens are labeled correctly.
- Surgical, anaesthetic, and nursing concerns for recovery are expressed before leaving theatre.

In paediatric surgical practice the parent is usually omitted from the safety check. Parental involvement in the Surgical Safety Checklist process has been recommended by WHO but is not universally accepted. This may reflect local concerns that parents are already stressed and that their involvement in the first stage of the Surgical Safety Checklist may add to their anxiety. There is also an unwritten concern that parental involvement will cause delay and add to staff anxiety in an already stressful environment.

## 2. Objectives

This prospective study was conducted to establish how theatre staff and parent(s) would accept parental involvement in the first stage of the three-stage WHO Surgical Safety Checklist performed in theatre before anaesthetic induction. We explored the question of whether parents and staff agreed with and welcomed parental involvement in the first stage of the three-stage WHO Surgical Safety Checklist.

## 3. Method

Following institutional approval at Our Lady's Children's Hospital, Crumlin, parents were given a leaflet detailing the nature of the normal Surgical Safety Checklist. Their approval to be involved was sought and they were asked to complete a survey before their child was discharged. The parents of all consecutive day-case patients with laterality issues were asked to participate in this study over a defined 6-week period. The patients ranged in age from two years to twelve years of age. Patients where laterality was not an issue, for example, umbilical hernia repair, were excluded from the study. There were no other exclusions, as all patients with laterality issues had an accompanying parent. In the first of the three stages of the WHO Surgical Safety Checklist a change was made. This was the standard first-stage check before induction of anaesthesia but this time with the parent present to assist in the confirmation of the child's identity, the operation to be performed, and the site of surgery and to confirm the correct consent. The parents were also asked to express any concern they may have. No parent refused to be involved in the checklist.

The parent remained with the child until after anaesthesia had been induced and was then accompanied out of the theatre suite by a theatre orderly.

Postoperatively, parents were asked the following questions.

1. Rate your involvement in the Surgical Safety Checklist.
2. Poor Average Good Excellent
3. Do you believe that your involvement improves patient safety? Yes No
4. Do you believe that the correct surgery was to be performed? Yes No
5. Should parental involvement in the Surgical Safety Checklist be mandatory for all children undergoing surgery? Yes No

All staff (nurses, anaesthetists, and surgeons) were also briefed as to the nature of the study and its objectives and asked to complete a questionnaire.

For staff the questions were as follows.

1. Rate the benefit of parental involvement in the Surgical Safety Checklist.
2. Poor Average Good Excellent
3. Do you believe that parental involvement in the Surgical Safety Checklist improves patient safety during surgery? Yes No
4. Do you feel that parental involvement in the Surgical Safety Checklist adds to the complexity of the process? Yes No

## 4. Results 

### 4.1. Parents

A total of 46 patients were admitted to the study. Four patients were omitted due to survey completion errors. 31% (13/42) and 69% (29/42) of parents rated their involvement in the Surgical Safety Checklist as good or excellent, respectively. All parents (100%, 42/42) considered their involvement as improving patient safety and 97.6% (41/42) considered that the site and procedure were correct. One parent expressed concern as the surgical team appropriately changed the consent prior to the operation to reflect a change of procedure. All parents (42/42) considered that parental involvement should be mandatory for all children undergoing surgery and none expressed any concern of added anxiety.

### 4.2. Staff

52.4% (22/42), 73.8% (31/42), and 28.6% (12/42) of nurses, surgeons, and anaesthetists rated parental involvement in the Surgical Safety Checklist as excellent while 47.6% (20/42) 26.2% (11/42) and 69% (29/42) rated it as good. 2.4% (1 anaesthetist) rated it as average. All staff believed that it improved patient safety and 100% of surgeons, 88% of nurses, and 76% of anaesthetists considered that parental involvement in the Surgical Safety Checklist did not add to the complexity of the process while the remainder felt that it was a justifiable addition.

## 5. Discussion

Risk management structures are now embedded in hospital practice and are accepted as a vital component of our efforts to prevent inadvertent surgical outcomes and wrong site surgery. The Surgical Safety Checklist is a relatively new concept aimed at ensuring that the correct patient is in theatre to have the correct procedure on the correct side and also to enable any team member to voice concerns about the planned procedure. In paediatric surgical practice however the parent is frequently excluded from this process. Of course informed consent has already been obtained and logically it would seem impossible for any error to occur. Yet it is the case that surgical errors are often due to a failure to communicate concerns or a continuation of the classic “Plan-Continue-Fail” scenario with potentially serious negative outcomes [4]. Generally parents are fully acquainted with the planned procedure but while they may have already expressed concerns this concern may not be relayed correctly or at all to the operating surgeon especially if a different staff member had taken consent. It is at this juncture that the latent marriage of human error and reliance on hospital systems, including the Surgical Safety Checklist, places the patient at most risk. Errors in communication occur with unacceptable frequency and are generally based on an assumption that concerns will be noted, addressed, and, more importantly, acted upon. While responsibility will always rest with the operating surgeon it seems prudent to provide further safeguards and ensure that all information will be passed on. We believed that it would be better to achieve this by ensuring that parents are able to express their approval or concerns at the time of induction in the presence of the operating team.

The reasons for not involving parents at this crucial stage may however underscore a belief that the Surgical Safety Checklist and consent process are robust enough and cannot possibly fail or that the institution and its staff believe that parents are already too stressed with the prospect of their child undergoing surgery. Parental involvement allows the parent to express concerns or questions which may not have been answered preoperatively or which may have come into the parent's mind after completion of the consent process. Paternalistic attitudes to minimise parental anxiety at the time of induction may explain why the Surgical Safety Checklist has not been more widely practiced in paediatric surgery. This study clearly demonstrates that parents valued their involvement in the Surgical Safety Checklist process and did so willingly with no reported added anxiety. It also demonstrated that all theatre staff did not feel parental involvement was intrusive to the operating list and that they too felt it should be a welcome part of the process.

## 6. Conclusion

Our results suggest that parents consider their involvement in the Surgical Safety Checklist as worthwhile and they consider that it should be mandatory. No parent expressed added anxiety from this involvement and 97% felt reassured that that correct procedure was to be performed. Similarly, staff responses confirmed the view that parental involvement improves patient safety and does not increase the complexity of the checklist process. This small, prospective study indicates that parents and staff alike consider that parental involvement in the Surgical Safety Checklist should be welcomed and encouraged, where local conditions allow it, to minimize the risks of wrong surgery or wrong site surgery.
